# Screens and Preschools: The Bilingual English Language Learner Assessment as a Curriculum-Compliant Digital Application

**DOI:** 10.3390/children11080914

**Published:** 2024-07-29

**Authors:** Hechmi Kilani, Ilia V. Markov, David Francis, Elena L. Grigorenko

**Affiliations:** 1 Department of Psychology, University of Houston, 3695 Cullen Boulevard, Room 126, Houston, TX 77204, USA; hechmi.kilani@times.uh.edu (H.K.); imarkov@cougarnet.uh.edu (I.V.M.); dfrancis@uh.edu (D.F.); 2 Texas Institute of Measurement, Evaluation and Statistics, University of Houston, 4349 Martin Luther King Boulevard, Houston, TX 77204, USA; 3 Center for Cognitive Sciences, Sirius University of Science and Technology, 354340 Sochi, Russia

**Keywords:** kindergarten readiness, preschool, tablet-based assessment, digital media use, children and screens, formative assessment, summative assessment, dynamic assessment, adaptive assessment, child psychology, education psychology

## Abstract

Background/Objectives: The increase in digital tools in early childhood education highlights the need for evidence-based assessments that support cognitive development and align with educational requirements and technological advances. This study contributes to the evaluation of the Bilingual English Language Learner Assessment (BELLA), designed to enhance early learning through curriculum-aligned tasks in preschool-aged children. Methods: Data were collected from 17 schools, including 506 preschool children, using a mixed-model approach to assess BELLA’s capacity to appraise early numeracy, literacy, science, and social/emotional development. Analyses included a three-way ANOVA to examine the effects of sex, age, and sub-domain on pass rates and mixed-effects models to evaluate interactions between age and domain. Results: The results indicated a significant effect of age on performance across all domains, with older children demonstrating higher pass rates (*p* < 0.0001). No significant gender bias was detected. The interaction between age and domain was also significant (*p* < 0.0001), suggesting domain-specific age-related performance trends, which aligns with internal validity requirements. Conclusion: These findings position BELLA within the growing body of literature on digital media use in early childhood assessment and education, highlighting its potential as a curriculum-compliant digital assessment tool that evaluates and supports cognitive development without a gender bias. This study contributes to the field by providing empirical evidence of BELLA’s effectiveness and suggesting future research directions, including the exploration of its bilingual (and potentially multilingual) applications and external validation against existing evidence-based assessments.

## 1. Introduction

The digital tablet (DT), a descendant of ancient clay tablets, is the peak of the transformative journey of writing, reading, and recording media. From archaic carvings in stone to modern digital screens, this lineage has affected education and learning by bringing new ways to record and disseminate information. DT use has become increasingly significant, and some research is unveiling how distinct it is from previous learning and assessment media (i.e., books, paper-based tests), displaying potential benefits in facilitating early cognitive development and learning. A recent systematic review of 35 studies supports the claim that DT use with interactive applications for early academic skills has benefits in typically developing children, especially within early mathematics. Overall, more favorable outcomes in mathematics, letter naming and writing, and phonological awareness were found in children using interactive applications [1]. Yet, innovations raise the need for adaptation and reform. The field of education falls short of that because of a lack of research on the dynamic learning processes enabled by DTs, a lack of co-use studies within the field of education, and a scarcity of educational applications that support precursors to higher-order cognitive skills [2,3,4].

The discourse is broad and multilayered when discussing the use of digital media in the classroom.

In fact, the body of literature relating to DT use as a learning tool seems to highlight the following elements: (1) early development is often not considered; (2) interactive touchscreen technology may provide a learning advantage for early academic learning; (3) studies overlook social/emotional development (particularly with children with Autism Spectrum Disorder); (4) studies disproportionately target mathematical and literacy skills; (5) the literature has a cultural bias and lacks non-Western samples; (6) studies overlook children’s co-use of tablets with caregivers; (7) studies focus on school use; (8) studies show mixed results, with either positive or non-significant effects for learning; and (9) DTs can be as effective as assessment methods in predicting outcomes as the traditional paper-and-pencil screenings [1,5,6,7]. Indeed, three recent systematic reviews [1,5,6] published in 2020, 2022, and 2024 converge on their findings, highlighting the above aspects of the literature, while more niche articles support the efficacy of tablet or computer use in lieu of traditional methods. The latter, in turn, may allow for quicker assessments while still accurately (as traditional assessments) predicting skills such as reading and letter knowledge [7].

This article sets the stage to address the topic of DT use in classrooms as a learning tool and a form of assessment, first by exploring the recent literature on digital media use in early development and second by overviewing digital-media-based classroom learning and curricula. Then, the Bilingual English Language Learner Assessment (BELLA), designed to cover gaps identified in the field of education and to be used in everyday practice as a kindergarten readiness assessment for children aged 3 to 5 years old, both mono-and bilingual, is introduced, and its first psychometric data are presented. The article focuses on the results of BELLA, as it is in its early stages of development, paving the way forward to future studies in varied cultural contexts (Arabic, Spanish, and Russian populations).

### 1.1. Digital Media and Early Development

Digital media have become ubiquitous in the lives of young children, where nearly all children under the age of eight have access to tablet or mobile devices, with the average time spent in front of a screen exceeding the recommended daily two hours of engagement [8]. However, DT and touchscreen devices have been recognized for their potential to revolutionize early childhood education and support lifelong learning by offering interactive and engaging ways for children to learn [9]. Additionally, children today are being introduced to digital technology at a younger age. Those aged 2 to 5 are able to more easily use a DT than they are able to tie their shoes [10], indicative of the easy and intuitive use of touchscreens. This ease of use is attractive, although important caveats need to be recognized.

First, children benefit from the co-use of digital media with a caregiver [2]. As early as 15 months of age, toddlers can learn from commercial media if parents watch with them and reteach the content to them by exploring it again during their interactions. By 24 months, children are able to learn words from video chats with a responsive adult or from interactive touchscreen applications [2]. In the classroom, it is crucial for the teacher to maximize benefits for children by taking into account that co-use is important [10] and therefore implement it in routines involving screen-based media. Second, there is an overwhelming number of DT applications that are marketed as educational. The Apple Store stood at 80,000 applications as of January 2015 [11] and had risen to more than 470 million applications by 2020 [12]. Android stores have 466 million applications [12]. Unfortunately, these applications that are marketed as such are untested and unregulated, and they are not designed to promote active, engaged, and socially interactive learning—conditions necessary to support learning and education in young children [12,13]. There is an evident empty pocket that needs investigation and the generation of practice-oriented recommendations; efforts should be exerted to create applications for young children that fit the standards of learning.

### 1.2. Digital Media Classroom Learning and Curricula

Early childhood education is increasingly characterized by the integration of digital media to foster and enhance learning and development. Meyer et al. [14] and the American Academy of Pediatrics [15] both highlight the potential benefits of DTs, specifying the need for high-quality content as well as adult supervision, and the importance of adult–child co-use. A study by Zaranis, Nicholas, Michail, Michail, and Stamatios [10] shows how mobile devices, and DTs specifically, can serve as significant educational tools. They offer interactive opportunities that are unmatched by traditional methods of learning. However, the authors highlight the fact that their efficacy is contingent on the educator’s ability to select and apply digital resources that align with pedagogical goals and are developmentally appropriate.

Additionally, the integration of DTs must consider classroom curricula. Originally, the National Education Goals Panel in 1991 laid the foundation for children’s learning in school, which covered five domains. The domains are physical well-being and motor development; social and emotional development; language and literacy development; cognition and general knowledge; and approaches to learning [16]. They show a prioritization of children’s overall physical and emotional well-being, communication skills, the ability to think and acquire knowledge, and the habits/behaviors expected for learning in a classroom [17]. These appear to be broad and invested in multiple aspects of child development. Curricula that focus on cognitive abilities are overlooked to focus on those designed to gauge balanced progress across multiple domains. Although they are designed to foster a broad range of cognitive skills, including creativity, this is not what is emerging from the literature [18]. Thus, DTs must be carefully used to cover all aspects of kindergarten readiness, including general progress and academic skills, but also creativity and more specialized cognitive skills involved in early numeracy, literacy science, and social/emotional development.

Building on these earlier insights and aiming to help with the identification of appropriate applications that educators can choose, Stamatios, Michail, Michail, Nicholas, and Nicholas [9] developed a rubric that takes into account four areas to consider: contents, design, functionality, and technical quality. Evaluating these four areas recognizes the transformative potential of digital media in education and highlights the necessity for tools that support a comprehensive learning experience that covers a wide range of cognitive and social/emotional skills. Robust educational applications are scarce, and educators need to be equipped to choose them. Tan, Kilani, Markov, Hein, and Grigorenko [4] contributed to this discussion and highlighted that assessment and learning tools need to foster and assess cognitive skills relevant to school curricula. Specifically focusing on Pre-K settings, it is important to be able to acknowledge the importance of not only general academic knowledge but also analytical, creative, and practical thinking skills, which are increasingly recognized as the skills of today and tomorrow [19]. BELLA, for example, is designed to align with the Texas Essential Knowledge and Skills guidelines, showcasing the possibility for educational applications to capture broad constructs and domains that are necessary for early education. Overall, the literature underlines the critical role of digital media in early childhood education, as well as the drawbacks associated with them. Digital media not only enhance traditional learning paradigms but also broaden the scope of assessment to include essential cognitive skills. When well designed following strict educational standards, educational applications are able to support the holistic development of young learners. By following strict standards and validating the psychometric abilities of applications such as those presented by Laura et al. [20] and Tan, Kilani, Markov, Hein, and Grigorenko [4], the field of early education can provide powerful tools to set up students for their developmental and educational journeys without overlooking the cognitive skills that are necessary for kindergarten readiness and that can be approached through early literacy, early numeracy, early science, and social/emotional readiness. Here, we present the preliminary validation process for BELLA after its design and implementation in American classrooms.

## 2. Materials and Methods

**Figure 1:**
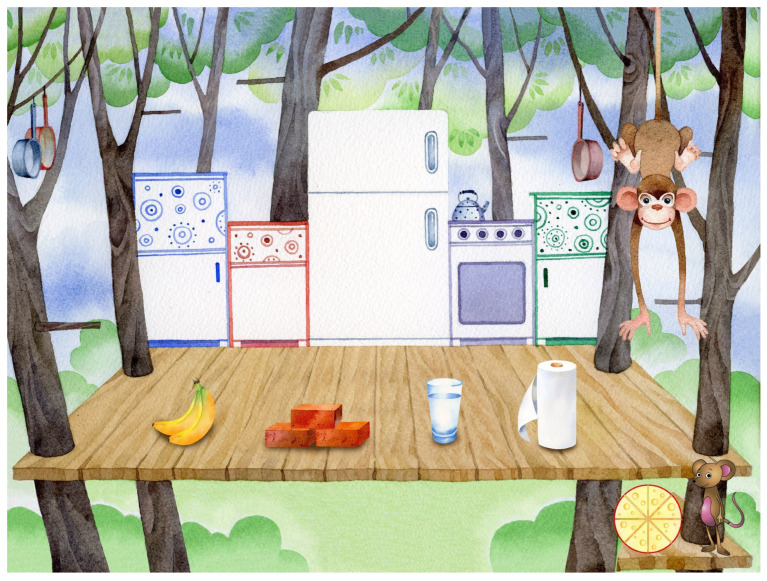
Item in the science domain assessing cognitive skill. It prompts, “Which of these things could help Monkey measure the area of her kitchen floor?”.

**Figure 2:**
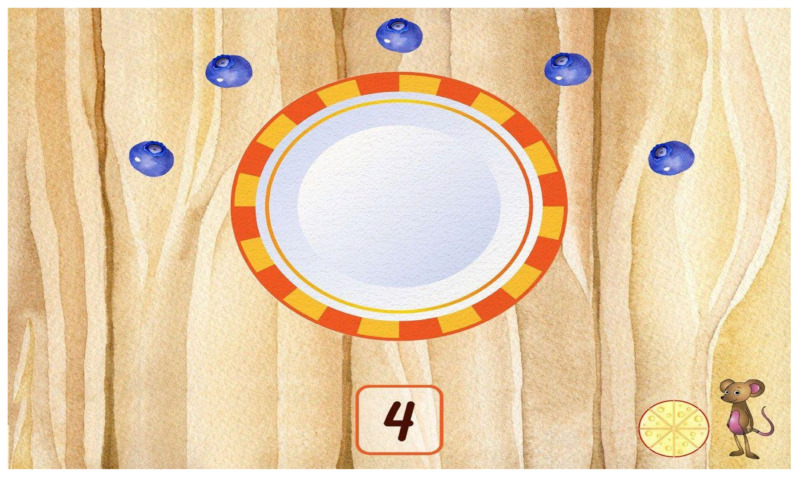
Item in the numeracy domain. It prompts the child to put a number of blueberries on the plate. The mouse is the companion throughout items, and the cheese wheel is a timer. The mouse provides feedback: it cheers when the answer is correct and shakes its head when it is not.

**Figure 3:**
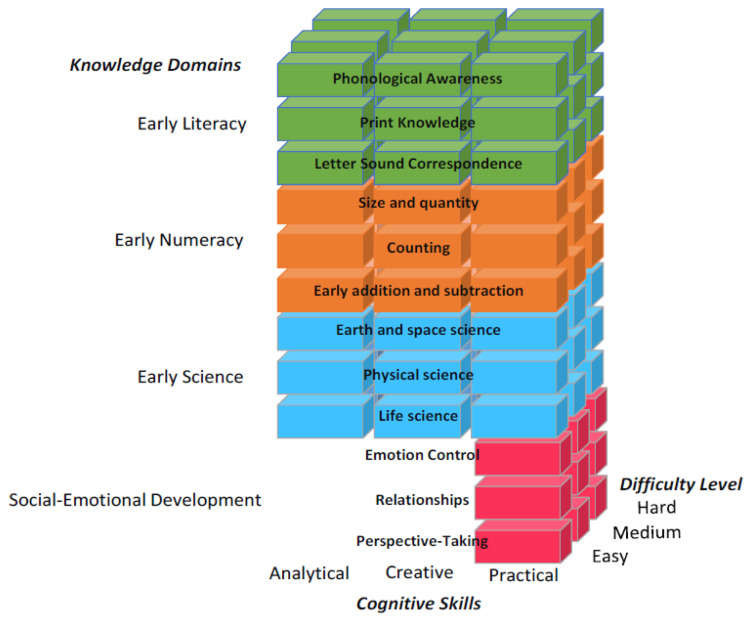
BELLA’s structure: knowledge domains and cognitive skills. Note: social/emotional items are only considered practical. There are no creative or analytical social/emotional items.

This study illustrates an application of a novel evidence-based assessment of kindergarten readiness in preschoolers. The goal is to verify that the application behaves as intended, that it specifically does not discriminate by gender, and that there is an expected age effect. The application, BELLA, is both an assessment and learning tool for preschool children aged 3 to 5 years old. It is a child-friendly application with storybook-like artwork and characters that accompany the child throughout the prompts (see Figure 1 and Figure 2). BELLA is designed for DT use only and follows a state-of-the-art methodology in its item design. Specifically, the application assesses four major domains: (1) early numeracy, (2) early literacy, (3) early science, (4) and social/emotional development. These domains are further split into 3 sub-domains each (see Figure 3) that vary in dimensions: difficulty and the cognitive skill assessed by the item. The former has three levels (easy, medium, hard), and the latter is split into analytical, creative, and practical cognitive skills. It is also designed with the theoretical frameworks of the USA national education standards [4], as well as the Texas Essential Knowledge and Skills (TEKS) guidelines, and addresses common standards across several states [4]. BELLA’s numerous items (over 700) can be used as summative or formative assessments and are well suited to tap into children’s analytical and practical reasoning and creativity, which are important aspects of child development. Additional information on BELLA’s domains, sub-domains, and cognitive skills is accessible in Tan et al. [4].

### 2.1. Data Collection

Data for the present study were collected across 17 schools from 26 March 2019 to 31 August 2022. Given constraints imposed by the COVID-19 pandemic on state and federal educational institutions, most of these schools were recruited from private preschool provider networks. Trained data collectors or trained teachers administered BELLA to children in their preferred language (the Spanish-language data are not included here; they are presented separately elsewhere). Each child completed at least one pilot path—a set of 33 items including 6 literacy, 6 math, 18 science, and 3 social/emotional items—lasting about 20 min. Children were removed from their classrooms and seated in a quiet room with the assessor to minimize distractions. Only a maximum of two children were assessed simultaneously by a tester, and minimal guidance was given to children who experienced some difficulty with selected items. Only one pilot path was administered per day. Children were given different pilot paths at each visit, up to three pilot paths per child. Children never took the same pilot path twice. Given the intent of this collection being used to aid in the validation of BELLA as an evidence-based and standardized tool, students were encouraged to take as many pilot paths as possible. Additionally, given the 20 min average that it takes to complete one pilot path, and given the age of the participants, we were able to stop a session whenever the child was not engaged or needed to take a break. In those instances, the session for the day was stopped, and the same pilot path was completed on another day.

### 2.2. Statistical Approach

The complex nature of the BELLA structure, reflecting several subtests that evaluate different knowledge and skill domains, makes the repeated-measures experiment framework the most optimal for the current study. Among several possible techniques conventionally used to carry out that type of analysis (uni- and multivariate repeated-measures ANOVA), the mixed-model approach is currently considered one of the most modern and flexible approaches [21] due to the following advantages: the ability to include observations with missing scores in the analysis [22], allowing for covariance analysis, and the ability to directly model the covariance structure [23], accounting for random variation at the between- and within-subject levels and using estimated generalized least squares. Relatedly, this framework can also be used in cases when data are suggested to not be normally distributed [24].

Besides the above-mentioned general advantages of the method, the mixed-model framework is specifically appropriate in this context due to the presence of missing scores and the heterogeneous structure of the sample, collected over several years from various schools at varied geographical locations. Based on the variety of school environments encountered during collection and the number of preschool students included in the analysis coupled with the complex domain structure, it would be reasonable to suspect random variation at both between- and within-subject levels, as well as a random group effect. Additionally, a preliminary description of the sample is provided, supplemented by a three-way ANOVA to examine the effects of sex, age, and sub-domain on pass rates. The independent variables were sex, age (4 levels, ranging from 3 to 6), and sub-domains (12 levels).

## 3. Results

### 3.1. Sample Characteristics

The final dataset (N = 506) comprised 288 males and 218 females. Age groups included 3-, 4-, 5-, and 6-year-olds, with 85, 233, 123, and 65 participants, respectively. There was an even distribution of sex within those age groups, except for the 6-year-olds, which included 2 females as opposed to 63 males. Additionally, four scores for each participant constituted their standardized mean compound scores for every knowledge domain of the test—literacy, math, science, and social/emotional. An overwhelming majority of participants were tested no more than twice (82.81%), using different pilot paths, with only 18 participants tested more than three times. Pass rates are used to score performance on BELLA. They are the average score of each participant across all items and pilot paths that were administered and are a reflection of correct answers to partial and non-partial items. Partial items are items with a two-step prompt structure: for example, prompt A is presented first, and prompt B is presented second as a continuation of the former. Non-partial items are items with a single prompt. Participants are able to make up to two errors per prompt. Upon two failures answering prompt A, prompt B is not presented, and a new item is presented. The descriptive characteristics of the study sample can be found in Table 1. Participants had a mean age of 4.33 years, and 43.1% were female. Most of the participants were 4 years old by a relatively large margin (46.05%), with the oldest and youngest age groups being the least populated (12.85% and 16.8%, respectively), possibly due to the sample reflecting the age group composition of a preschool environment.

Additionally, the study sample was grouped by cognitive skill and sub-domain (Table 2 and Table 3, respectively). Overall, the results show that average pass rates gradually increase as age increases and that genders score at similar averages. When grouped by sub-domain, the trend is conserved for both genders and all age groups, with all sub-domains having an average pass rate above 50 except for phonological awareness, which lies at 46.6. The highest average pass rate belongs to the relationships and emotional control sub-domain, with a score of 80.4. All cognitive skills stood at a pass rate above 50, with the lowest being the creative skill at 52.3. BELLA displays early signs of validity, as children seem to perform similarly according to gender and increasingly better according to age. An exception is the oldest group (i.e., 6-year-olds).

### 3.2. Preliminary Exploration

#### 3.2.1. Sex × Sub-Domain × Age

A three-way ANOVA was conducted to examine the effects of sex, age, and sub-domain on pass rates. There was a significant main effect of age on pass rates (*F*(3, 5501) = 64.68, *p* < 0.0001) and a significant main effect of sub-domain (*F*(11, 5501) = 15.2, *p* < 0.0001). There was no significant main effect of sex on pass rates (*F*(1, 5501) = 1.14, *p* = 0.2867). Additionally, interactions between the variables were analyzed. The interaction term for age × sub-domain was significant (*F*(33, 5501) = 1.97, *p* < 0.0001). However, the interaction between sex and age was not significant (*F*(3, 5501) = 1.39, *p* = 0.2447), nor was the interaction between sex and sub-domain (*F*(11, 5501) = 0.79, *p* = 0.6475). The three-way interaction between sex, age, and sub-domain was also not significant (*F*(33, 5501) = 0.68, *p* = 0.9134). The overall model was significant (*F*(95, 5501) = 10.27, *p* < 0.0001), with an R-squared of 0.150, indicating that approximately 15% of the variability in pass rates can be explained by the model.

#### 3.2.2. Age × Sub-Domain

A two-way ANOVA was conducted to examine the effects of age and sub-domain on pass rates, ignoring the sex variable. The model was significant (*F*(47, 5549) = 20.16, *p* < 0.0001) and explained approximately 14.58% of the variance in pass rates (*R*² = 0.145829). There was a significant main effect of age on pass rates (*F*(3, 5549) = 73.84, *p* < 0.0001) and a significant main effect of sub-domain (*F*(11, 5549) = 48.26, *p* < 0.0001). The interaction term for age x sub-domain was also significant (*F*(33, 5549) = 2.92, *p* < 0.0001). This suggests that the effect of age on pass rates varied across the different sub-domains. This implies that both age and the specific sub-domain independently affect the pass rate, and the interaction between these two factors also has an influence. This may suggest that different age groups have different performance levels across the sub-domains.

#### 3.2.3. Sex × Sub-Domain

A two-way ANOVA was conducted to examine the effects of sex and sub-domain on pass rates, ignoring the age variable. The model was significant (*F*(23, 5573) = 26.57, *p* < 0.0001) and explained approximately 9.88% of the variance in pass rates (*R*² = 0.098807). There was a significant main effect of sex on pass rates (*F*(1, 5573) = 4.31, *p* = 0.0379) and a significant main effect of sub-domain (*F*(11, 5573) = 52.23, *p* < 0.0001). The interaction term for sex x sub-domain was not significant (*F*(11, 5573) = 0.82, *p* = 0.6217). This suggests that the effect of sex on pass rates did not vary across the different sub-domains. In conclusion, while both sex and the specific sub-domain independently affect the pass rate, the interaction between these two factors does not have a significant influence. This may suggest that regardless of the sub-domain, the pass rate does not significantly differ between sexes.

#### 3.2.4. Summary of ANOVAs

Analyses suggest that pass rates vary significantly across age groups and across sub-domains. However, pass rates did not significantly vary between sexes. There was also an interaction effect between age and sub-domains, which was significant with *F*(33, 5501) = 1.97, *p* < 0.0008, suggesting that the relationship between age and pass rates differed across sub-domains.

Further analysis was also conducted for each interaction effect. For the interaction between age and sub-domain, the significant interaction effect persisted (*F*(33, 5501) = 2.92, *p* < 0.0001). For the interaction between sex and sub-domain, the interaction effect was not significant (*F*(11, 5573) = 0.82, *p* = 0.6217). We can infer that age and sub-domain have relatively large effects on the pass rates, with age having the largest effect. The interaction between age and sub-domain also seems to have a meaningful effect on the pass rates. The effect of sex, as well as the interaction between sex and sub-domain, appears to be smaller and non-significant. In conclusion, both age and sub-domain significantly impact pass rates, and the effect of age on pass rates changes across different sub-domains. Sex does not significantly affect pass rates, and its interaction with the sub-domain is also non-significant.

### 3.3. Mixed Models’ Results

**Figure 4:**
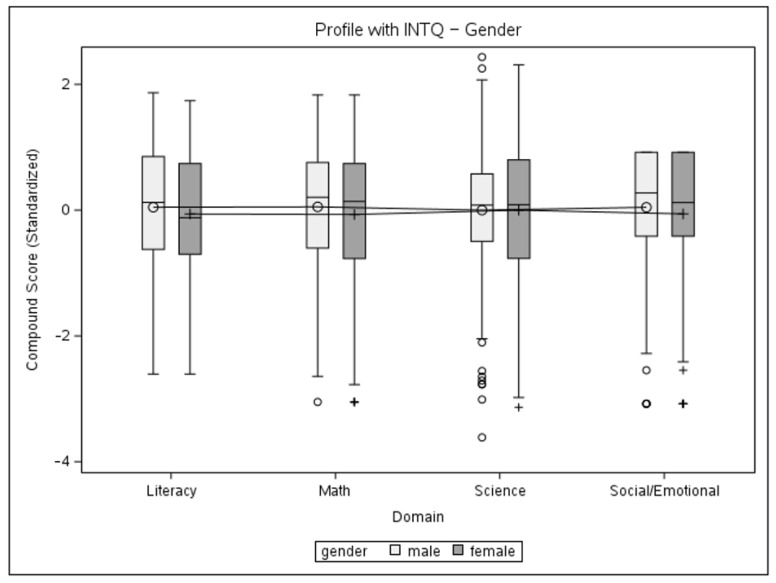
Profile plot of gender and domain interaction with interquartile ranges.

**Figure 5:**
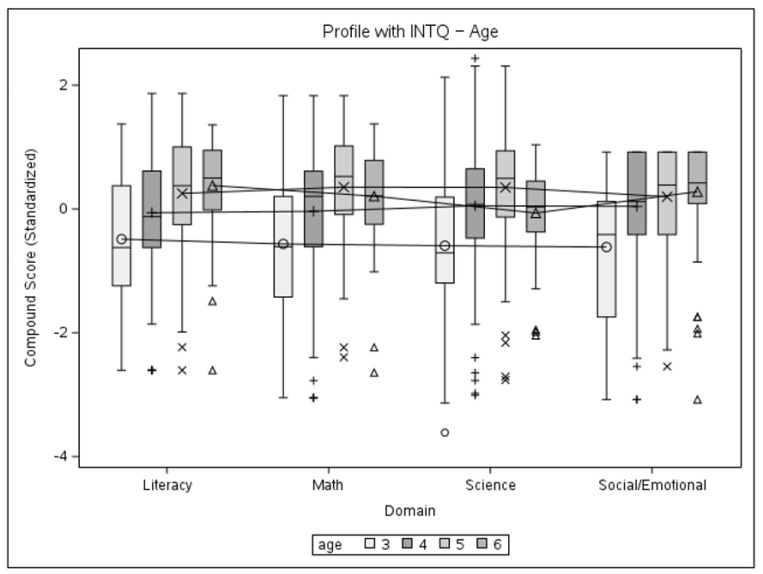
Profile plot of age group and domain interaction with interquartile ranges.

The data from the present sample were further used to conduct a mixed-model multivariate analysis, with the dependent variable being the standardized domain compound score, with four observations for each child. The first model includes the main effects of domain and gender and their interaction while permitting the variance to be different between genders. The second mixed model is similarly fitted to look at the main effects and the interaction between the domain and age group. The two-tailed significance level was set at α = 0.05. Initially, an unstructured variance–covariance matrix was used for both models. The gender model did not achieve significance for main effects or interactions, so no follow-up tests were conducted. A significant main effect of age (*F* = 22.44, *p* < 0.0001) and an interaction effect (*F* = 5.9, *p* < 0.0001) were registered for the age-group model. Choosing a compound symmetry type of covariance matrix for the age-group model did not yield a significant loss of fit. Thus, this pattern is deemed satisfactory for an explanation of the variance in the data. The solutions for the fixed effects of gender and age-group models are given in Table 4, and profile plots with interquartile ranges for the models can be seen in Figure 4 and Figure 5. Based on the significance of the interaction, follow-up tests were conducted in the form of complex comparisons between cell means to verify the significance of performance differences between age groups. The type of contrasts used necessitated the use of Scheffe’s adjustment to determine significance. Estimates for contrasts are given in Table 5.

**Figure 6:**
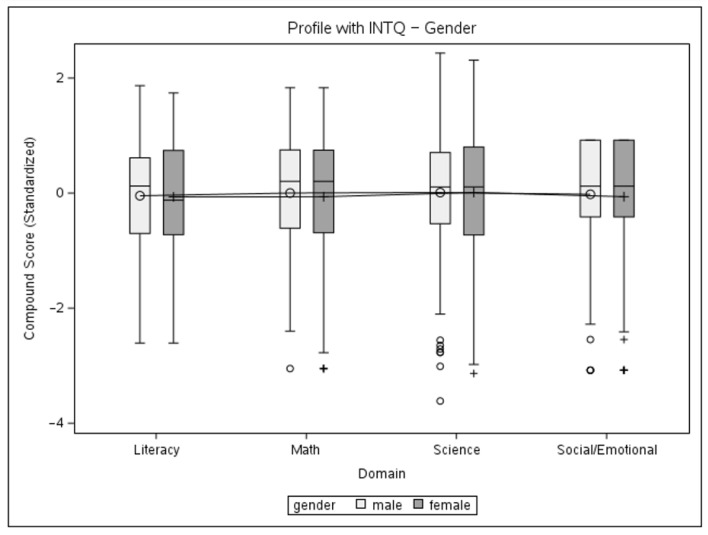
Profile plot of gender and domain interaction for ages 3–5 with interquartile ranges.

**Figure 7:**
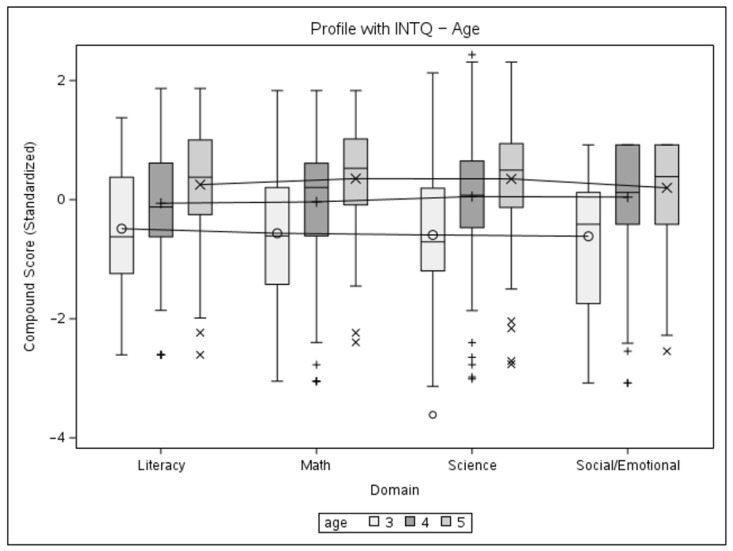
Profile plot of age and domain interaction for ages 3–5 with interquartile ranges.

The results of the second mixed model brought our attention to an irregular score pattern seen in the 6-year-old age group, which might be explained by the smaller group size and several unobservable factors that would lead to a child remaining within the school system at that age. A second pair of models was fitted after excluding this group to estimate its effect on the results (Table 6). The structure of the first analysis was replicated on the sub-sample, with two mixed models using an unstructured variance–covariance matrix for the levels of interest, followed by a single model using a compound symmetry structure. Based on the significance of the main effect of the age group, follow-up tests were conducted in the form of marginal mean estimates (Table 7) at α = 0.05. Profile plots with interquartile ranges for the models can be seen in Figure 6 and Figure 7.

The results of the mixed models confirm the adequate psychometric properties of BELLA, specifically its construct validity, in the following aspects: no significant difference between domains and gender groups, but a significant effect of age with an upward trend in performance, with a slight irregularity seen for the 6-year-old group. Follow-up comparisons for the compound symmetry age-group model were designed to explore the source of the difference between the levels of that factor. The first two sets of comparisons (3 + 4 vs. 5 + 6) confirm the significance of the upward trend in performance. However, the last set of comparisons (3 + 4 + 5 vs. 6) shows that children in the oldest age group do not perform significantly better than their younger counterparts, which is unexpected from the developmental standpoint, with science as the only domain not demonstrating a negative estimate for the contrast. Possible reasons for this might be a small group size and the data coming from a limited number of schools, in addition to several factors not possible to observe during the collection effort that would lead to children staying in the preschool system for longer.

The second pair of models with the 6-year-old age group excluded show a similar pattern of results. The interaction between the domain and age was no longer significant, while the main effect of age and the upward trend in performance were retained. Better fit indices for the second set of models did not deviate from what can be explained by a smaller sample size. Follow-up marginal mean estimates show significance for the 3- and 5-year-old groups, in accordance with them being the oldest and youngest groups of the sub-sample. The profile plots demonstrate a clearer upward trend of increasing performance with age, which only slightly varied by domain.

## 4. Discussion

This evaluation of the Bilingual English Language Learner Assessment, BELLA, highlights its potential as a pivotal tool in early childhood education that is evidence-based and corresponds to US federal and state standards of school readiness. It specifically aims to enhance critical preschool skills, and our findings agree with the systematic review by Griffith, Hagan, Heymann, Heflin, and Bagner [1], which stresses the broad efficacy of digital applications in supporting early learning domains. BELLA achieves this by not only evaluating specific academic domains but also incorporating methods for cognitive assessment. The results show that the application displays appropriate psychometric properties by not discriminating by gender and by showing increased performance as age increases. Additionally, the levels of difficulty of items are appropriate, as the pass rates are relatively high and meet a priori expectations of what children should be able to do at a particular age. BELLA seems to fit with the required characteristics of effective early tools for educational assessment and practice/development. It allows for adult mediation as well as child-independent use with minimal supervision/interference in early learners, given its tablet format. BELLA’s design and the study’s results align with curriculum requirements as well.

BELLA finds its niche within the digital media and early education literature. Specifically, its conception and purpose are to allow for a comprehensive assessment of young children’s knowledge and skills. It can be used as a way of measuring the efficacy of teaching methods, course plans, and so on. The uniqueness of BELLA also lies in that it is available in multiple languages and would allow for a more equitable assessment of ability and knowledge for children who do not speak English at home or for children in less represented countries and cultures, covering gaps highlighted by Griffith and their team [1], as well as in the systematic review by Alotaibi [5].

Overall, the accessibility, curriculum compliance, format, and co-use potential of the assessment align with recommendations emerging within the field [2,25]. This synergy that is provided between the digital tool quality and the interactive learning experiences is crucial for maximizing the developmental benefits of digital media. A well-designed assessment and instruction digital tool can significantly contribute to early childhood educational settings, and it is crucial that such tools are evidence-based and steer clear of particular biasing assumptions [3], which BELLA achieves. BELLA is an instrument that is capable of assessing a broad and comprehensive spectrum of cognitive skills, which addresses an important gap in early educational assessments and modern-day requirements [4]. It fulfills the need for an innovative assessment tool that capitalizes on the advantages of the current status of digital technology. Comparisons to other instruments discussed in Tan, Kilani, Markov, Hein, and Grigorenko [4] demonstrate that BELLA is one of the more comprehensive assessment applications in the field.

Regarding our findings, the implications suggest that, while creating applications for young children, educators, curriculum developers, application creators, policymakers, and artists should consider an approach that invites every one of them to create or utilize a tool that maximizes its advantages and that is compliant with educational requirements. Currently, too many applications are marketed as suitable for early education yet do not display the robust characteristics of an educational tool. BELLA, however, was created by a diverse team of educators, researchers, and art designers—allowing it to support traditional learning objectives and preparing children for their future academic life and challenges by fostering critical thinking and practical skills from an early age. The application is designed for children aged 3 to 5 years old, and the results confirm the successful achievement of this requirement. Children who are older and still in preschool show decreased performance as a group, which will need further exploration to unveil the reasons why this is the case. Potential reasons include underlying developmental conditions or environmental challenges that prevent the child from progressing into kindergarten and being kept behind in preschool. BELLA indeed arises from the need to cover gaps in the literature, capitalizing on its capability to address cognitive skills and a broad range of knowledge domains; to be used with diverse and international populations; and to be used individually or with a caregiver, at home or at school.

## 5. Limitations and Future Directions

Limitations to our study include our sample size and a lack of diversity among the young children recruited, which prevented the cross-comparison with Spanish speakers and their performance with similar items to the English ones. In an increasingly diverse educational environment, tools that operate in multiple languages are necessary, and efforts are being implemented to broaden the potential pool of BELLA users across the globe, with versions in languages such as Russian and Arabic currently being developed. Moreover, BELLA’s longitudinal performance is yet to be uncovered, as these results represent a summative use of the application rather than its formative use throughout the school year. The diversity of educational settings needs to be increased, as our sample may be biased toward the children of families that typically frequent private preschools. Ideally, BELLA would be investigated on a larger scale within the public school system. While the internal validation process has been initiated with the present paper, one more crucial aspect of validity must not be neglected: the external validation of BELLA with current off-the-shelf assessments. Nevertheless, BELLA is garnering great potential for diverse cultural contexts.

Future works with BELLA will target its application in a variety of cultural contexts. Special attention will be given to the children’s interactions with the application, caregivers, and tasks, adding an important behavioral component to the research. Cross-cultural studies are to be conducted between languages (e.g., multiple Spanish-speaking countries) to verify that BELLA is not biased toward dialects prevalent in the United States or toward Western culture.

## 6. Conclusions

In conclusion, our study contributes to the literature by affirming the value of digital assessment and learning tools in early childhood education and their potential to revolutionize traditional teaching and assessment methods and modernize current digital-based assessments. Researchers [1,2,3,4,25] agree on the necessity for ongoing innovation, specifically for early education, and argue for novel research and multidisciplinary collaboration to fully harness the educational benefits of digital technologies and tablets for young learners. We concur, and BELLA is our attempt to add to the growing library of well-designed and needed and expected assessment tools.

## Figures and Tables

**Table 1:** Descriptive statistics of the sample and average pass rates.

Analysis Variable: Pass Rate				
Age	Sex	N	Mean	Std Dev
3	female	45	53.6773	17.9981
	male	40	51.7828	14.4356
4	female	119	63.3162	14.5783
	male	114	63.7005	12.4611
5	female	52	69.4444	12.0181
	male	71	68.8912	14.409
6	female	2	54.2576	2.12132
	male	63	65.7999	11.2099

**Table 2:** Descriptive statistics of scores by sub-domain and age.

Analysis Variable: Pass Rate				
Sub-Domain	Age	*N*	Mean	Variance
Counting	3	69	49.9217	1307.26
	4	202	64.1788	920.61
	5	113	71.0167	848.674
	6	62	59.4594	508.845
Early Addition and Subtraction	3	85	44.651	1010.53
	4	233	50.9922	1088.15
	5	123	59.7793	854.07
	6	65	57.1026	558.605
Earth and Space Science	3	85	55.0993	550.643
	4	233	67.3418	405.841
	5	123	70.6373	280.649
	6	65	61.9641	135.609
Emotional Control; Emotion Recognition	3	59	74.5763	1928.7
	4	170	67.5294	2011.32
	5	103	80.0647	1463.99
	6	61	89.8907	724.803
Empathy	3	73	55.4338	1818.98
	4	208	76.5854	1142.87
	5	118	77.0682	1145.47
	6	64	78.3333	1006.53
Letter/Sound Corres.	3	70	50.3429	900.049
	4	203	62.1149	645.804
	5	113	70.8098	535.11
	6	62	84.0118	326.548
Life Science	3	85	47.2902	448.169
	4	233	53.9798	341.19
	5	123	61.8297	271.126
	6	65	59.065	163.66
Phonological Awareness	3	59	34.6017	679.61
	4	170	47.5624	694.334
	5	103	53.2737	745.158
	6	61	44.515	541.652
Physical Science	3	85	58.6085	578.198
	4	233	69.2574	295.213
	5	123	71.7937	352.835
	6	65	64.2509	156.535
Print Knowledge	3	85	52.4353	1565.07
	4	233	60.8774	1184.71
	5	123	68.1721	930.227
	6	65	71.4192	673.376
Relationships; Emotional Control	3	85	61.4706	1603.8
	4	233	82.5603	917.626
	5	123	87.6039	623.72
	6	65	83.8462	603.379
Size and Quantity	3	68	65.288	790.269
	4	194	72.3288	628.153
	5	96	80.9239	392.449
	6	60	82.7292	243.988

**Table 3:** Descriptive statistics of scores by cognitive domain and age.

Analysis Variable: Pass Rate					
Cognitive Skill	Age	*N*	Mean	Variance	Std Dev
Analytical	3	85	54.3529	479.504	21.8976
	4	233	66.6527	271.325	16.472
	5	123	73.8463	248.543	15.7652
	6	65	75.2179	215.457	14.6785
Creative	3	85	44.9531	273.975	16.5522
	4	233	54.3781	239.614	15.4795
	5	123	59.4256	276.64	16.6325
	6	65	42.0446	70.6016	8.40248
Practical	3	85	57.5566	348.652	18.6722
	4	233	68.0166	250.741	15.8348
	5	123	72.8625	226.715	15.0571
	6	65	75.927	209.233	14.4649

**Table 4:** Solutions for fixed effects and fit statistics for mixed-effects models for the total sample.

Solution for Fixed Effects					Fit Statistics
Gender * Domain—Unstructured					
Effect	Num DF	Den DF	F Value	*p*	−2 Res Log Likelihood
Domain	3	1512	0.02	0.9974	AIC
Gender	1	504	1.45	0.2284	AICC
Gender * Domain	3	1512	0.8	0.4951	BIC
Age * Domain—Unstructured					
Effect	Num DF	Den DF	F Value	*p*	−2 Res Log Likelihood
Domain	3	1506	1.08	0.3563	AIC
Age	3	502	22.44	<0.0001	AICC
Age * Domain	9	1506	5.9	<0.0001	BIC
Age * Domain—Compound Symmetry					
Effect	Num DF	Den DF	F Value	*p*	−2 Res Log Likelihood
Domain	3	1506	0.99	0.3972	AIC

Note: * denotes interaction.

**Table 5:** Cell mean contrasts for the age-group model for the total sample.

Contrast Levels	Estimate	Standard Error	DF	*t* Value	*p*
Lit −3 + 4 vs. 5 + 6	−1.1784	0.187	1506	−6.3	<0.0001 *
Math −3 + 4 vs. 5 + 6	−1.1622	0.187	1506	−6.21	<0.0001 *
Science −3 + 4 vs. 5 + 6	−0.8264	0.187	1506	−4.42	<0.0001 *
Soc/Em −3 + 4 vs. 5 + 6	−1.0519	0.187	1506	−5.62	<0.0001 *
Lit −3 + 4 vs. 5	−1.0512	0.2134	1506	−4.92	<0.0001 *
Math −3 + 4 vs. 5	−1.3072	0.2134	1506	−6.12	<0.0001 *
Science −3 + 4 vs. 5	−1.2393	0.2134	1506	−5.81	<0.0001 *
Soc/Em −3 + 4 vs. 5	−0.9701	0.2134	1506	−4.55	<0.0001 *
Lit −3 + 4 + 5 vs. 6	−1.4328	0.3242	1506	−4.42	<0.0001 *
Math −3 + 4 + 5 vs. 6	−0.8722	0.3242	1506	−2.69	0.0072
Science −3 + 4 + 5 vs. 6	−0.0007	0.3242	1506	0	0.9984
Soc/Em −3 + 4 + 5 vs. 6	−1.2153	0.3242	1506	−3.75	0.0002

Note: * Significant under Scheffe’s adjustment for multiple comparisons.

**Table 6:** Solutions for fixed effects and fit statistics for mixed-effects models for ages 3–5.

Solution for Fixed Effects					Fit Statistics	
Gender * Domain—Unstructured						
Effect	Num DF	Den DF	F Value	*p*	−2 Res Log Likelihood	4710.1
Domain	3	1317	0.63	0.5926	AIC	4750.1
Sex	1	439	0.18	0.6711	AICC	4750.5
Sex * Domain	3	1317	0.17	0.9183	BIC	4831.8
Age * Domain—Unstructured						
Effect	Num DF	Den DF	F Value	*p*	−2 Res Log Likelihood	4599.7
Domain	3	1314	0.35	0.7912	AIC	4659.7
Age	2	438	30.96	<0.0001	AICC	4660.8
Age * Domain	6	1314	1.16	0.3268	BIC	4782.4
Age * Domain—Compound Symmetry						
Effect	Num DF	Den DF	F Value	*p*	−2 Res Log Likelihood	4686.8
Domain	3	1314	0.4	0.7523	AIC	4690.8
Age	2	438	35.6	<0.0001	AICC	4690.8
Age * Domain	6	1314	1.12	0.3473	BIC	4699

Note: * denotes interaction.

**Table 7:** Marginal means for the mixed model of age and domain interaction for ages 3–5.

Age	Estimate	Standard Error	DF	*t* Value	*p*
3	−0.5654	0.07815	438	−7.24	<0.0001
4	−0.0014	0.0472	438	−0.03	0.9759
5	0.2876	0.06496	438	4.43	<0.0001

## Data Availability

Data can be obtained upon request from the corresponding author.
